# The impact of security countermeasures on human behavior during active shooter incidents

**DOI:** 10.1038/s41598-022-04922-8

**Published:** 2022-01-18

**Authors:** Runhe Zhu, Gale M. Lucas, Burcin Becerik-Gerber, Erroll G. Southers, Earl Landicho

**Affiliations:** 1 Sonny Astani Department of Civil and Environmental Engineering, Viterbi School of Engineering, University of Southern California, Los Angeles, CA 90089 USA; 2 USC Institute for Creative Technologies, Los Angeles, CA 90094 USA; 3 Sol Price School of Public Policy, University of Southern California, Los Angeles, CA 90089 USA

**Keywords:** Civil engineering, Psychology and behaviour

## Abstract

Active shooter incidents represent an increasing threat to American society, especially in commercial and educational buildings. In recent years, a wide variety of security countermeasures have been recommended by public and governmental agencies. Many of these countermeasures are aimed to increase building security, yet their impact on human behavior when an active shooter incident occurs remains underexplored. To fill this research gap, we conducted virtual experiments to evaluate the impact of countermeasures on human behavior during active shooter incidents. A total of 162 office workers and middle/high school teachers were recruited to respond to an active shooter incident in virtual office and school buildings with or without the implementation of multiple countermeasures. The experiment results showed countermeasures significantly influenced participants’ response time and decisions (e.g., run, hide, fight). Participants’ responses and perceptions of the active shooter incident were also contingent on their daily roles, as well as building and social contexts. Teachers had more concerns for occupants’ safety than office workers. Moreover, teachers had more positive perceptions of occupants in the school, whereas office workers had more positive perceptions of occupants in the office.

## Introduction

Human safety is one of the most important considerations for building design and management^1,2^. Indeed, a wide range of natural and anthropogenic emergencies could impose risks on buildings, including fires, earthquakes, and floods^3^. Unfortunately, in the U.S., active shooter incidents present an increasing threat. According to the Federal Bureau of Investigation (FBI), an active shooter is an individual actively engaged in killing or attempting to kill people in a confined and populated area^4^. There were approximately 15 active shooter incidents in the U.S. annually from 2000 to 2018, of which the majority occurred in various built environments, led by commercial buildings (43.7%) and educational buildings (20.6%)^5^. Sadly, the risk of active shooter incidents is rising. 28 active shooter incidents occurred in the U.S. in 2019 alone, which caused 97 fatalities and 150 injuries^6^. Compared with other types of emergencies, active shooter incidents have several distinctive characteristics. First, while natural hazards follow certain intrinsic rules of evolution, shooters can have specific targets and strategically respond to the ongoing situation. For example, shooters can scan the surrounding environment to choose their targets, or might demonstrate behaviors that are not expected, which increases the uncertainty of active shooter incidents^7,8^. Second, the duration of active shooter incidents is usually very short: 70% of incidents ended within 5 min and 60% ended before police arrived^4^. The high uncertainty and limited response time during active shooter incidents show the urgent need to take appropriate measures to mitigate the safety risks during active shooter incidents.

Concerning human safety during active shooter incidents, previous studies have mainly focused on two stages: the preparation stage and the response stage. To improve people’s preparedness for active shooter incidents, providing training and drills has been considered as an effective approach^9^. People can familiarize themselves with appropriate actions and safety resources through training and drills, and hence gain confidence in responding to active shooter incidents^10^. Pre-event guidelines (e.g., the “Run, Hide, Tell” guidance in the U.K. and the “Run, Hide, Fight” guidance in the U.S.^11^) have also been proposed to equip people with necessary knowledge and skills for protective behaviors. More recently, different training paradigms, such as multi-option approaches (e.g., avoid, deny, defend), have been examined in addition to the traditional lockdown drills to better prepare people for active shooter incidents^12^. As for the response stage, various factors can play critical roles. A critical determinant is available information and guidance during the event. When the information about a shooter’s location is provided, people are more likely to respond appropriately and have higher chances to survive^13^. The impact of situational information is also correlated with social influence: if information is provided, people tend to be less likely to form groups with others^14^. Moreover, building attributes can affect people’s responses. For instance, corridor and exit width have been found to have significant influences on people’s safety when an active shooter incidents occurs^8^. Apart from the research efforts, public and governmental agencies have also published guidelines on emergency preparedness, response, and management to improve human safety during active shooter incidents. For example, the Interagency Security Committee released a guideline, in which the importance of preparedness (e.g., establishing threat assessment teams) and training was highlighted^15^. New York City Police Department (NYPD) recommended developing response plans, conducting drills, and implementing security countermeasures (e.g., controlling access to buildings and installing bullet-resistant windows) to better prepare for active shooter incidents^16^. Additionally, Federal Emergency Management Agency (FEMA) released several guidelines for building safety design in preparation for active shooter incidents and other types of attacks^17–19^. While these guidelines involve a wide range of aspects, we chose to focus on the recommendations related to building design due to the following reasons. First, buildings, such as workplaces and schools, usually have high crowd density, which makes the occupants suitable targets and thus increases the motivation of active shooters^20^. Buildings provide the essential conditions that affect people’s chances of survival. Many building attributes, including corridor configuration and exit location have a direct impact on human behavior and safety during emergencies^21,22^. Second, the effectiveness of building design-related countermeasures, such as access control, need to be further assessed with regards to their influence on human behavior^9^. Some building design-related countermeasures aimed for improving building security may have negative effects on human safety when an active shooter incident occurs. In fact, previous studies have shown that human behavior is one of the most critical determinants of building emergency outcomes^23^. For instance, during the 2003 Rhode Island station nightclub fire, even though multiple emergency exits were present, people swarmed to the main exit and ignored other ones, resulting in dozens of casualties^24^. Similarly, the design of emergency signage depends on people’s perception and recognition (e.g., color and location of signage)^25,26^. With this in mind, we aim to empirically examine building design-related countermeasures for active shooter incidents with an emphasis on their influence on human behavior.

### The impact of human-building-emergency interactions on human behavior

Far from being an isolated concept, human behavior is interconnected with buildings, surrounding people, and emergencies^27^. One study revealed that compared with office buildings, people in residential buildings tended to have longer pre-evacuation time^28^. Such impact of building types can be attributed to the theory of situated cognition: the context in which an event occurs affects people’s interpretation, which in turn affects the outcomes^29^. The theory of situated cognition was originated in the field of educational psychology and has been applied in various domains. In education, the theory of situated cognition represents an approach to learning that emphasizes the importance of the social and cultural context in which learning occurs^30^. For example, when learning a language, the context in which a person learns the language could affect their understanding of the language (e.g., how the words are used in a specific social interaction)^31^. In emotion perception, it was shown that structural feature of a face is not the only determinant of emotion perception, and the context in which the face is included is also an important factor^29^. In social psychology, researchers revealed that cognition and action are outcomes of dynamic interactions between a person and an environment^32^. Moreover, in the context of building emergencies, how people respond can vary according to the contextual environments. For example, architectural visual access has an impact on people’s route choices and evacuation performance^33^. People’s spatial knowledge and familiarity with the building can also affect their route-choice decisions^34,35^. While the impact of context has been considered in prior studies, investigations of human behavior during active shooter incidents across different types of buildings are still limited. Moreover, due to the contextual dependency of human behavior, the effectiveness of countermeasures is also likely to vary in different buildings. This lack of cross-building studies represents an important gap in this research area. Apart from buildings, surrounding people can also affect human behavior during building emergencies. For instance, people often consider the presence of others and decide to follow or avoid the crowd while evacuating to their desired destinations^36^. Furthermore, with regards to emergency types, Haghani and Sarvi^37^ claimed that human behavior depends on emergency attributes. Perry and Lindell^38^ also posited that findings about human behavior cannot be directly transferred from one emergency to another. As active shooter incidents have only investigated in a few studies, the patterns of human behavior during active shooter incidents -across different types of buildings- are examined in this study.

### The impact of psychological ties on human behavior

Apart from human-building-emergency interactions, psychological ties also influence human behavior during building emergencies^39^. It has been found that people with close psychological ties (e.g., families, friends, and coworkers) attempt to stay together, and even re-enter the building to search for missing members^39,40^. Drury et al.^41^ conducted virtual evacuation experiments with participants of the same or different social identities. Their results showed that if informed that the surrounding crowd attends the same university or supports the same football club, participants would behave more cooperatively than competitively. In another study, it was found that if participants identify themselves or others as Muslims, they would feel safer with increased crowd density at the Hajj^42^. Moreover, psychological ties do not only exist among people with the same social identity or pre-existing relationships but can also emerge ad hoc during building emergencies. For example, people may form groups based on a shared identity and develop a feeling of “us” instead of “them”, which leads to the feeling of responsibility or obligations to care for those that they might be implicitly or explicitly responsible for and exhibit helping behavior even if they are strangers^43^. The impact of psychological ties has been applied, elaborated, and interpreted in different contexts^44^, whereas their influence during active shooter incidents remains unexplored. Therefore, we aim to investigate if/how people perceive the shooter and others differently based on the building and social contexts.

### The impact of daily roles on human behavior

People’s roles affect their behavior during building emergencies as well. Canter et al.^45^ proposed role-rule theory, which postulated that during building emergencies, human behaviors directly relate to their roles in daily life and rules associated with the role. More recently, Shiwakoti et al.^46^ conducted a survey with staff members and passengers in a train station. The results showed that staff members were more likely to behave proactively due to their familiarity with evacuation procedures. On the contrary, passengers tended to rely on the instructions from staff members. Tancogne-Dejean and Laclémence^47^ also found that for vulnerable people, the presence of first responder teams take on two roles: to reassure and to advise. Several specific behaviors during building emergencies can be attributed to role-rule theory: staff members are more likely to provide help and guide occupants to evacuate appropriately^48^, and teachers often serve as an actuator for students to start responding to building emergencies^49^. Under high crowd density, people may form lines and follow a leader, who oftentimes has had a leader role in daily life^50^. Moreover, the impact of daily roles is not solely determined by people’s level of knowledge and skills, but also their emotional attachment to others. For example, staff managers often extend their roles and assume additional responsibilities for those who work under them in the event of an emergency^51^. Along this line, we aim to investigate if/how people’s responses to active shooter incidents are related to their daily roles, and if/how such relationship further affects the effectiveness of security countermeasures.

### Research methods for studying human behavior during building emergencies

Due to legal and moral reasons, it is impractical to study human behavior during real-world building emergencies. Accordingly, many alternative methods have been used, such as case studies, interviews and surveys, laboratory experiments and drills, and animal experiments^52^. While these methods are commonly used, they bear several intrinsic limitations. Case studies are highly dependent on the available data of real-world emergency incidents. Interviews and surveys rely on respondents’ subjective answers and suffer from memory bias. Laboratory experiments and drills lack the capability of evoking sufficient emotional arousals; hence participants may not behave the same as they would during real-world emergencies. Moreover, animal experiments are criticized in that the results may not be transferrable to humans^52^. In recent years, the development of virtual reality technology has provided a promising method for behavioral studies. The main advantage of a virtual environment is that it provides researchers with the flexibility to manipulate and control variables in a non-intrusive and realistic environment^53,54^. Virtual environments have been increasingly used to study various human behaviors during building emergencies, including examining different building designs and social influence^55–57^. The main concern about adopting virtual environments, however, is ecological validity (i.e., whether the findings in virtual environments can be comparable to the real world). To tackle this issue, several previous studies applied psychological (e.g., emotional arousal, sense of presence) and physiological (e.g., heart rate variability, skin conductance) measurements and demonstrated that participants had similar behavioral, cognitive, and psychophysiological reactions in virtual and real environments^58,59^.

### Present study

Driven by the above-mentioned motivations, we conducted virtual experiments to investigate people’s responses to an indoor active shooter incident. A preliminary experiment was conducted to validate the ecological validity of our virtual environments, using the same buildings and shooting scenario as the present study^60^. Three research questions were examined in the present study:

- If/how do security countermeasures, which are usually implemented to improve building security, affect human behavior during active shooter incidents?
- If/how do people perceive and respond to active shooter incidents differently according to the building (i.e., different building types) and social (i.e., different social groups) contexts?
- If/how do people perceive and respond to active shooter incidents differently according to their daily roles (i.e., occupational backgrounds)?

## Methods

### Virtual environment

**Figure 1:**
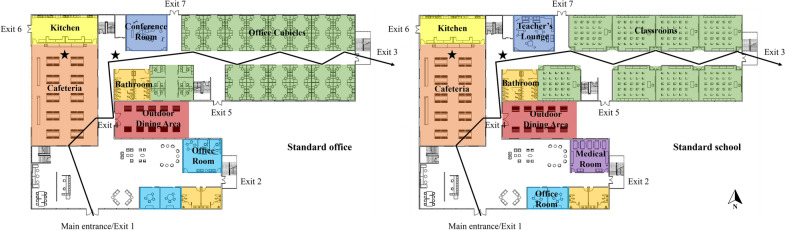
First-floor layouts of the standard office (left) and school (right). Stars denote participants’ starting locations: one in the cafeteria and one in the hallway. Arrowed lines denote the shooter’s movement trajectory.

An office building and a school building were geometrically modeled to simulate an active shooter incident in indoor environments, as these two buildings have been most frequently targeted by shooters^5^. The two buildings were both based on a Revit model provided by an architecture, design, planning and consulting firm. A licensed architect further reviewed the models to ensure that they were representative of real-world office and school buildings. The first-story layouts of the two buildings are shown in Fig. 1. Both buildings had 7 exits, 5 staircases, and a cafeteria and a kitchen exactly in the same locations. To avoid any potential confounding factors, building size and structure were kept identical between the two buildings. Several manipulations were adopted merely to represent the context of an office and a school without changing the building layout or complexity: in the office, a conference room replaced the teacher’s lounge, an office room replaced the medical room, and office cubicles replaced classrooms in the school.

To assess the impact of security countermeasures on participants’ responses to the active shooter incident, a list of countermeasures was identified by conducting a comprehensive literature review and focus group interviews with 15 building designers/engineers, security experts, and law enforcement professionals^9,61^. The countermeasures were implemented and resulted in another version of the office and school (hereafter referred to as enhanced buildings as opposed to standard buildings, which had no countermeasures). The description and implementation of the countermeasures are presented in Table 1. In total, four different virtual environments were included in the experiment, namely (1) standard school, (2) standard office, (3) enhanced school, and (4) enhanced office.

**Table 1:**
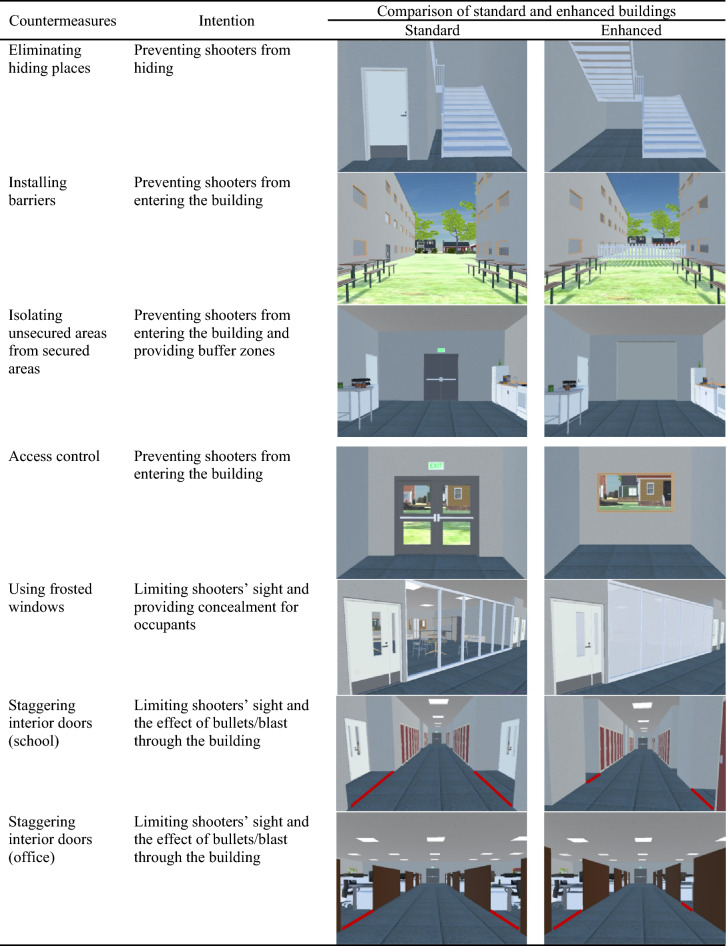
Security countermeasures included in the virtual environments.

**Figure 2:**
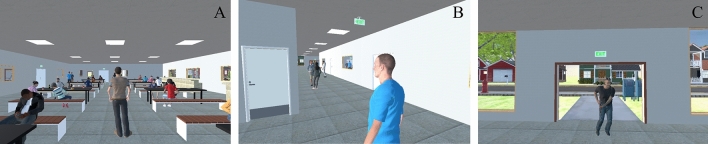
Virtual occupants and the virtual shooter. (**A**): occupants from the view of the starting location in the cafeteria. (**B**): occupants from the view of the starting location in the hallway. (**C**): shooter entering the building from the main entrance (see Fig. 1 for the starting locations and main entrance).

Non-player characters (NPCs) as building occupants were included in the virtual environments to increase realism and represent the natural effect that crowd behavior has during emergency situations. Each virtual environment contained 81 building occupants, which was determined considering the tradeoffs between the level of realism and computational burden. 25 occupants were initially in the cafeteria, 12 were in the hallways, 4 were in the teacher’s lounge/conference room, and the remaining 40 were in the office cubicles/classrooms. It is noteworthy that while we kept the occupants’ appearance, gender, and clothing the same across different conditions to avoid potential confound, we intentionally modeled them to look relatively young so that they can reflect the corresponding social contexts (office workers in the office and students in the school). Figure 2A, B illustrate some occupants at their initial locations. The occupants were given different response times ranging from 0 to 10 s when the shooting started, which were determined based on the occupants’ distance to the shooter and were reviewed by a former FBI agent to ensure realism. After occupants started to respond to the shooting, they would navigate to preprogrammed destinations. We distributed the destinations among different exits and hiding places to reflect the natural difference between the standard and enhanced buildings and avoid biasing participants’ decisions. For example, if barriers were implemented in the outdoor dining area, naturally occupants could not evacuate there, and instead they were assigned to other exits according to their initial locations. Other than these, occupants’ behaviors remained the same among different conditions.

The shooter was represented by a male character (Fig. 2C), as most active shooter incidents in the U.S. involved a single shooter and the majority of them were males^5^. The shooting started 10 s after the beginning of the experiment and ended after 105 s. The shooter would first enter the building from the main entrance and go to the cafeteria. Then he would move along the hallway and leave the building via Exit 3. During the movement, the shooter would shoot the occupants that are visible to him. Once being shot, occupants would fall onto the ground and stay still, however, no blood would appear to avoid violent and graphic content. It is important to note that at the beginning of the experiment, participants were not in close proximity to the shooter and could not directly see the shooter. Nevertheless, participants may encounter the shooter during the experiment depending on the directions they decide to go. While this information was not provided to the participants, the shooter could not shoot them during the experiment so that all participants could complete their trials.

### Apparatus and simulations

The virtual environments were initially created for in-person experiments using VR headsets and controllers. However, due to the COVID-19 pandemic, we had to modify the virtual environments as WebGL applications and deployed them on GitHub. Such a setup allowed participants to access the virtual environments remotely in a web browser using laptops/desktops, keyboards, and mice for social distancing. Unity game engine was used to develop the virtual environments. Sound effects, including occupants’ chatting and panic sound and shooting sound (represented a semi-automated assault rifle, which was recommended, reviewed, and approved by a former FBI agent) were added to the virtual environments. Moreover, participants’ locations in the virtual environments and the corresponding timestamps were updated and recorded twice every second during the experiment.

### Experiment design

This study used a 2 (design: standard or enhanced) × 2 (order: office before school or school before office) × 2 (building: office or school) mixed-subjects design, where the latter factor was within-subjects. Starting location (cafeteria or hallway) for the first trial was also randomized, and the other location was then used for the second trial. As a result, there were 8 experiment groups, as shown in Table 2. Each group consisted of two trials and each participant was randomly assigned to one of the groups. Between the two trials, building design was kept the same, whereas building type, order, and starting location were different. The metrics to measure participants’ responses included their response time and decision. The response time was defined as the time participants spent from the start of the experiment to the moment that they completed the last action (e.g., evacuating the building via an exit or hiding at a place). Decision referred to whether participants chose to run, hide, or fight, and what specific destination (e.g., an exit or hiding place) they chose. Response time and decision were determined by reviewing video recordings of the experiment and the recorded data. Moreover, to examine participants’ perceptions and considerations during the experiment, subjective questions were asked in the surveys.

**Table 2:** Experiment groups.

Group	Design	First trial	Second trial
1	Standard	Office, cafeteria	School, hallway
2		School, hallway	Office, cafeteria
3		Office, hallway	School, cafeteria
4		School, cafeteria	Office, hallway
5	Enhanced	Office, cafeteria	School, hallway
6		School, hallway	Office, cafeteria
7		Office, hallway	School, cafeteria
8		School, cafeteria	Office, hallway

### Participants

A total of 162 adults (36.73 ± 11.14 years-old on average, 71 males and 91 females, 79 middle/high school teachers and 83 office workers) participated in the study. 5 participants were excluded due to technical issues with the virtual environments, or the participants did not finish the experiment. All participants received a $20 Amazon Gift Card as monetary incentives after the study. Participants were recruited nationwide via social media platforms (e.g., Facebook, Craigslist) and emails, and their occupations were verified by asking them to provide a work email address or an ID. All participants reviewed the informed consent and agreed to participate in the experiment. This study was approved by the University Park Institutional Review Board (UPIRB) of University of Southern California. Since minors are considered to be more vulnerable or at-risk, we only recruited adults in this study to limit risks. All experiments were performed in accordance with relevant guidelines and regulations.

### Procedure

A Zoom meeting was used to connect participants with the experimenter. At the beginning, participants reviewed an information sheet, which described that the objective of this study was to examine how building designs influence human responses during emergency scenarios. Following the informed consent, participants completed a training session, in which they followed the experimenter’s instructions to get familiar with different actions (i.e., turn, walk, run, and crouch) in the virtual environments. The shooter was not included in the training environment in order not to reveal the type of emergency they would experience, and participants could not directly fight against the shooter to ensure all participants experienced the same shooting scenario during the same amount of time. That being said, participants were not informed that they could not fight either, hence some participants may still choose to confront the shooter. Accordingly, the “fight” decision in this study denotes those participants confronted or approached the shooter. The training took place in a virtual outdoor space, which was different from the experiment environment. Next, participants completed a pre-experiment survey, which asked their demographic information and positive and negative emotions measured with the Positive Affect and Negative Affect Scale (PANAS) from 1 (not at all) to 5 (extremely)^62^.

**Figure 3:**
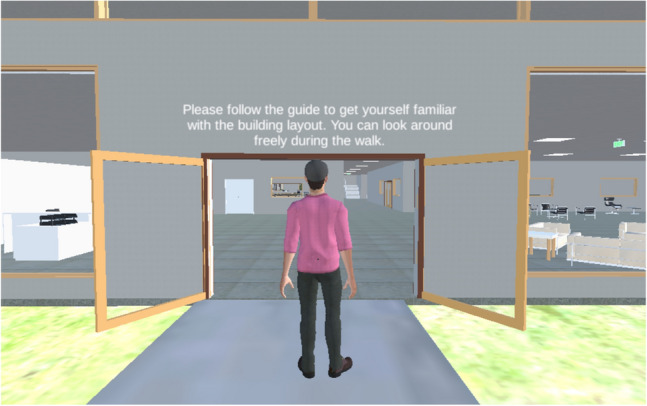
Virtual tour guide for the building tour.

Participants then familiarized themselves with the building by following a virtual tour guide, as shown in Fig. 3. The virtual guide was preprogrammed to follow a given trajectory to show participants different sections of the building. The tour took approximately 4 min. The participants were asked to familiarize themselves with the building without pointing out countermeasures implemented in the enhanced building to avoid potential confounding effects. Subsequently, participants responded to the active shooter incident in the same building (i.e., first trial). Upon the completion of the first trial, participants responded to a mid-experiment survey, which asked their perceptions of the shooter (from 1 as “neutral” to 5 as “worst thing ever”) and occupants (from 1 as “extremely negative” to 5 as “extremely positive”), whether they considered different factors (shooter and occupant behavior, exits, hiding places, and occupant safety) when responding to the active shooter incident from 1 (strongly disagree) to 5 (strongly agree), and positive and negative emotions measured with PANAS. Specifically, with regards to the perception of the shooter and occupants, we asked participants to rate their high-level perceptions about the presence, appearance, and behavior of the shooter and occupants.

After completing the mid-experiment survey, participants went through a second trial, as shown in Table 2. Similarly, they followed the virtual tour guide to familiarize themselves with the building and responded to the active shooter incident (i.e., second trial). Finally, participants completed a post-experiment survey, which asked the same questions as in the mid-experiment survey, in addition to their sense of presence (i.e., the subjective experience of being in one place or environment, even when one is physically situated in another) measured with the presence questionnaire from 1 (not at all) to 7 (extremely)^63^, their familiarity with the building and shooting from 1 (not at all) to 5 (extremely), and previous experience with active shooter incidents or drills. The total duration of the experiment was approximately 45 min. Participants were thanked and dismissed after completing the experiment.

### Analysis

Repeated measures analysis of covariance (ANCOVA) was mainly used for the analysis. Since each participant experienced two trials with different building types and starting locations (see *Experiment design*), building type was entered as the within-subjects factor and starting location was entered as the covariate. Between-subjects factors included design, occupation, and order. Dependent variables in the repeated measures ANCOVA included participants’ response time, decisions, and subjective perceptions. To analyze decisions, we considered participants’ choices among run, hide, and fight. Because the order recommended by authorities for surviving an active shooter incident is run, then hide, then fight^64^, a continuous variable was created where higher scores are further down this recommended order of actions (1: run, 2: hide, 3: fight). Moreover, to reveal if any individual difference variables affected participants’ response time and decisions, we conducted linear regression analysis with participants’ age, gender, and previous experience with active shooter incidents as independent variables. The significance level was set at 0.05 and the marginal significance level was set as 0.10. All the data analysis was conducted using the SPSS 25 software.

## Results

### Ecological validity

**Figure 4:**
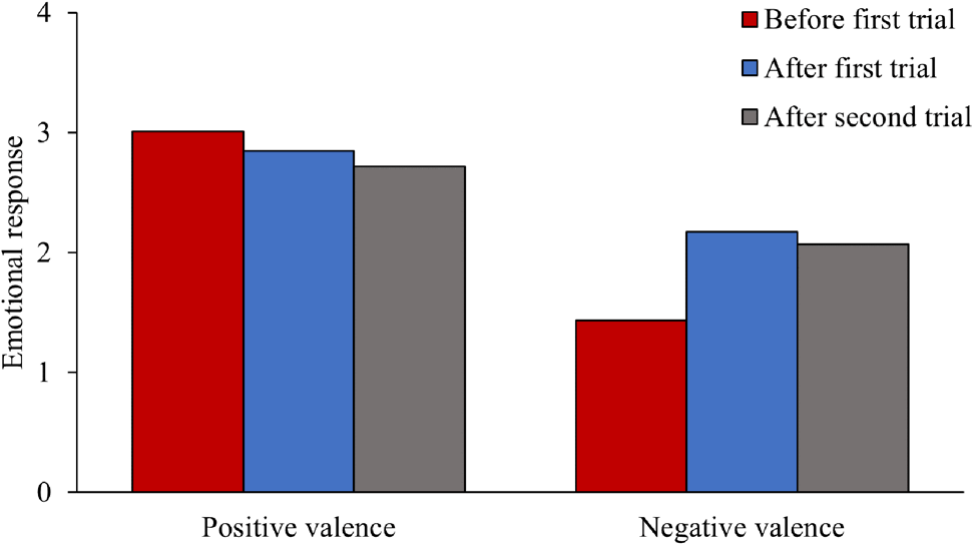
The effect of valence × time interaction on participants’ emotional arousals.

Before analyzing participants’ responses to the active shooter incident, we examined their emotional responses and sense of presence to assess the ecological validity of our virtual environments. For emotional response, a 2 (valence: positive or negative) × 3 (time: before the first trial, after the first trial, or after the second trial) × 2 (design: standard or enhanced) × 2 (order: experienced the office before school or experienced the school before office) × 2 (occupation: office workers or teachers) factorial ANCOVA was conducted, with valence and time as within-subjects factors, design, order, and occupation as between-subjects factors, and location (started in the hallway or kitchen) as the covariate. The results revealed a significant valence × time ($F_{2, 296}$ = 8.346, $p = 0.001, \eta_{p}^{2}$ = 0.053) interaction, such that participants’ positive emotions decreased consistently during the two trials, and negative emotions increased significantly during the first trial and decreased slightly during the second trial (Fig. 4). This result is in accordance with our previous findings^60^, which showed that the virtual environments evoked a large increase of participants’ negative emotions. Given the complex nature of our experiment design, there were also inscrutable valance × time × design ($F_{2, 296}$ = 9.319, $p < 0.001, \eta_{p}^{2}$ = 0.059) and valence × time × occupation ($F_{2, 296}$ = 3.018, $p = 0.062, \eta_{p}^{2}$ = 0.02) interactions. There were also other main effects and lower-order interactions, which were less relevant as they collapsed across time/valence or were qualified by the above effects (see *Analysis of participants’ emotional response* in the Supplementary Information).

We asked participants’ sense of presence only once (asking them to estimate across both trials) in the post-experiment survey, thus we conducted a 2 (design) × 2 (order) × 2 (occupation) between-subjects factorial ANCOVA, with location as the covariate. There was a marginally significant main effect of order ($F_{1, 148}$ = 3.036, $p = 0.084, \eta_{p}^{2}$ = 0.02), such that the participants experiencing the office before school (M = 135.73) had a lower sense of presence than those experiencing the school before office (M = 142.05), perhaps because a school shooting is more psychologically engaging. More importantly for our validation, sense of presence averaged 4.63 across all participants on a scale from 1 to 7. The results indicated that our virtual environments induced significant emotional arousals and adequate sense of presence for the participants, which supports validity of the following results.

### Response time and decision

We first analyzed whether participants’ individual differences affected their response time, hence a linear regression analysis was conducted with response time as the dependent variable and participants’ age, gender, and previous experience with active shooter incidents as independent variables. The results showed that response time in the office was significantly affected by participants’ age (*β* = 0.192, $p$ = 0.018), such that younger participants had a shorter response time than older participants. Next, to evaluate how participants’ response time was affected by the experimental conditions, we conducted a 2 (building: office or school) × 2 (design) × 2 (order) × 2 (occupation) factorial ANCOVA, with building as the within-subjects factor, design, order, and occupation as between-subjects factors, and location as the covariate. The results revealed a marginally significant main effect of building ($F_{1, 148}$ = 3.437, $p = 0.066, \eta_{p}^{2}$ = 0.023), such that participants spent less time in the office (M = 38.95 s) than school (M = 43.73 s). This effect was qualified by an interaction with order (building × order, $F_{1, 148}$ = 11.837, $p = 0.001, \eta_{p}^{2}$ = 0.074): participants had a shorter response time in the office if they experienced the school before office (M = 36.66 s for the office, M = 49.07 s for the school). Moreover, although it was a smaller difference, they had a shorter response time in the school if they experienced the office before school (M = 41.23 s for the office, M = 38.39 s for the school). As building and order relate to participants’ first and second trials, this result demonstrated that participants spent less time responding to the shooting in the second trial, and this was especially true when the office was second. Likewise, a marginally significant main effect of design ($F_{1, 148}$ = 2.799, $p = 0.096, \eta_{p}^{2}$ = 0.019) was found, such that participants in standard buildings (M = 38.84 s) had a shorter response time compared with those in enhanced buildings (M = 43.84 s). This result indicated that, with the implementation of countermeasures in enhanced buildings, participants might have needed more time to reach a desired destination. Given the complex nature of our experiment design, there was also an inscrutable design × order × occupation ($F_{1, 148}$ = 3.149, $p = 0.078, \eta_{p}^{2}$ = 0.021) interaction (see *Analysis of participants’ response time* in the Supplementary Information).

**Figure 5:**
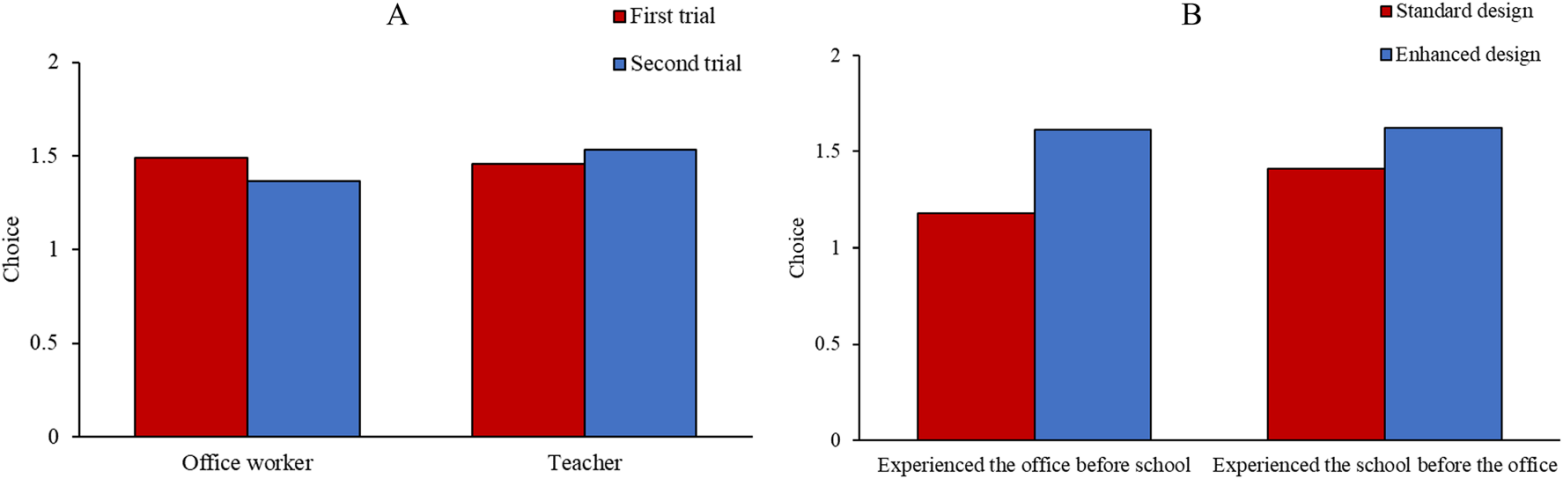
Participants' choices among run, hide, and fight. (**A**): the effect of building × order × occupation interaction. (**B**): the effect of design × order interaction.

To analyze participants’ choices among run, hide, and fight, we first examined the impact of individual differences by conducting the same linear regression analysis as for response time. The results showed that participants’ decisions in the office were significantly affected by their previous experience with active shooter incidents (*β* = 0.167, $p$ = 0.039), such that in the office, participants who had previous experience with active shooter incidents were more likely to hide in place. Next, we conducted another ANCOVA for participants’ decisions with building as the within-subjects factor, design, order, and occupation as between-subjects factors, and location as the covariate. This analysis revealed a significant building × order × occupation ($F_{1, 148}$ = 4.206, $p = 0.042, \eta_{p}^{2}$ = 0.028) interaction. Since there were a total of only five participants chose to fight (4 office workers and 1 teacher), the interaction effect revealed that office workers scored lower and thus chose “run” more often in the second trial than the first, whereas teachers scored higher in the second trial and thus chose to “hide” more in the second trial than the first, as shown in Fig. 5A. This result indicated that, with repeated trials, people would fall more into the expected or normative patterns associated with their occupation (e.g., teacher to stay to protect students). Main effects of design ($F_{1, 148}$ = 23.718, $p < 0.001, \eta_{p}^{2}$ = 0.138) and order ($F_{1, 148}$ = 3.058, $p = 0.082, \eta_{p}^{2}$ = 0.02) were found and qualified by a marginally significant design × order ($F_{1, 148}$ = 2.733, $p = 0.098, \eta_{p}^{2}$ = 0.018) interaction: participants were more likely to run instead of hiding in standard buildings than in enhanced buildings, especially for those experiencing the office before school (Fig. 5B). This result suggested that the implementation of certain countermeasures (e.g., access control) might have impeded participants’ ability to run in enhanced buildings.

Next, we examined participants’ decisions at a more fine-grained level by looking into their choices of specific exits or hiding places. As no within-subjects effect was found for participants’ decisions of run, hide, or fight in the above analysis, the two trials were collapsed here. Specifically, we created another continuous variable to denote whether a choice was made 0, 1, or 2 times by a participant across the two trials. For each exit and hiding place, a 2 (design) × 2 (order) × 2 (occupation) between-subjects factorial ANCOVA was conducted, with location as the covariate. The results are shown in Table 3. First, as Exits 4, 5, 6 and 7 were removed in enhanced buildings, we investigated participants’ choice of evacuating via Exit 3 (at the end of the hallway). The result revealed a significant main effect of design, such that participants in enhanced buildings (M = 0.61) evacuated via Exit 3 more frequently than those in standard buildings (M = 0.18). Second, there were main effects of design and order on participants’ choice of hiding in the kitchen, such that participants in enhanced buildings (M = 0.25) or those experiencing the school before office (M = 0.2) chose to hide more in the kitchen than those in standard buildings (M = 0.03) or experiencing the office before school (M = 0.07). Similarly, in enhanced buildings, participants chose to hide more often under staircases (M = 0.17 for standard buildings, M = 0 for enhanced buildings) or in the conference room/teacher’s lounge (M = 0.04 for standard buildings, M = 0.14 for enhanced buildings). The above results suggest that the implementation of countermeasures in enhanced buildings shaped participants’ choices of specific exits and hiding places, and the order of experiencing the office and school also played a role in participants’ choices.

**Table 3:** Effects of design and order on participants’ choices (see Fig. 1 for the building layout).

Dependent measure	Effect	F	*p*-value	$\eta_{p}^{2}$
Evacuating via Exit 3	Design	20.005	< 0.001	0.119
Hiding in the kitchen	Design	16.409	< 0.001	0.1
	Order	5.272	0.023	0.034
Hiding under staircases	Design	15.261	< 0.001	0.093
Hiding in the conference room/teacher’s lounge	Design	4.713	0.032	0.031

### Subjective responses

We also examined participants’ subjective responses to the active shooter incident in terms of perceptions of the shooter and other occupants, reports of how much their responses were influenced by the shooter and other occupants, and finally, the degree to which participants reported considering other factors (i.e., exits, hiding places, occupant safety) in making their decisions. For each subjective response, we again conducted a 2 (building) × 2 (design) × 2 (order) × 2 (occupation) factorial ANCOVA, with building as the within-subjects factor, design, order, and occupation as between-subjects factors, and location as the covariate.

**Figure 6:**
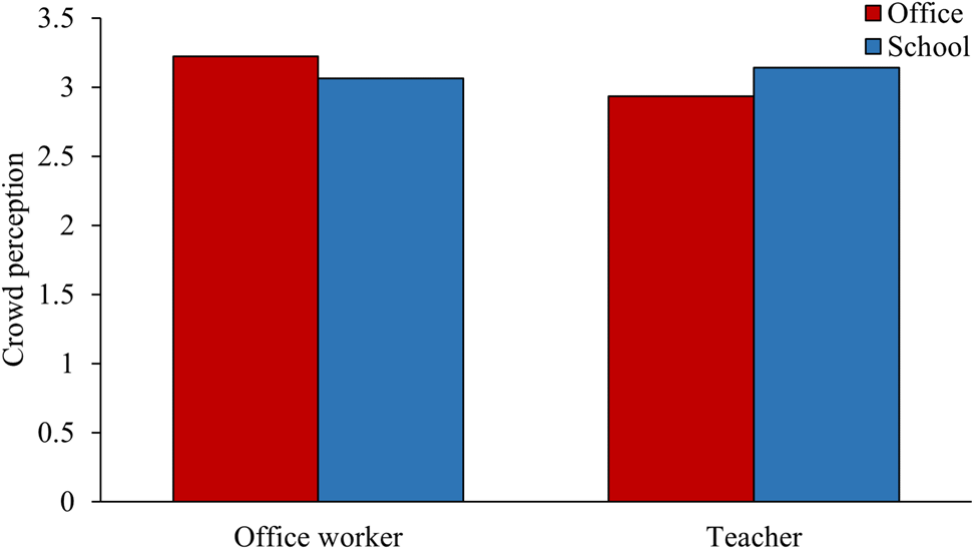
The effect of building × occupation interaction on participants’ perceptions of other occupants.

First, we analyzed participants’ perceptions of the shooter and other occupants. Main effects of occupation ($F_{1, 148}$ = 4.665, $p = 0.032, \eta_{p}^{2}$ = 0.031) and order ($F_{1, 148}$ = 3.103, $p = 0.08, \eta_{p}^{2}$ = 0.021) were found for participants’ perceptions of the shooter: office workers (M = 4.51) and participants experiencing the school before office (M = 4.48) perceived the shooter more negatively, compared with teachers (M = 4.2) and participants experiencing the office before school (M = 4.23), who might have possibly had more exposure to (because of traits associated with their occupation) or less judgement of (because office is not as cruel of a target as school) the shooter. Considering perceptions of other occupants, a marginal main effect of building ($F_{1, 148}$ = 3.564, $p = 0.061, \eta_{p}^{2}$ = 0.024) was found, but it was qualified by a significant building × occupation ($F_{1, 148}$ = 5.852, $p = 0.017, \eta_{p}^{2}$ = 0.038) interaction: office workers had more positive perceptions of other occupants in the office, whereas teachers had more positive perceptions of other occupants in the school (Fig. 6). Even though the occupants had the same appearance in the office and school, participants liked the occupants better when they were in their own occupational context.

**Figure 7:**
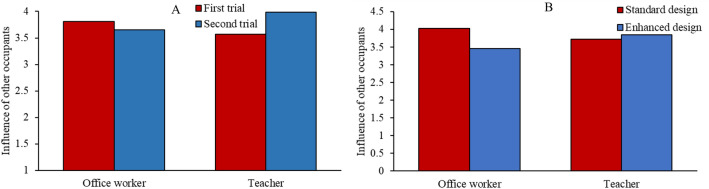
Influence of other occupants on participants' responses. (**A**): the effect of building × order × occupation interaction. (**B**): the effect of design × occupation interaction.

We next evaluated participants’ reports of how their responses were influenced by the shooter and other occupants. For influence the shooter had on responses, given the complex nature of our experiment design, inscrutable interactions (see *Analysis of shooter influence on participants’ response* in the Supplementary Information) were found for design × order × occupation ($F_{1, 148}$ = 3.221, $p = 0.075, \eta_{p}^{2}$ = 0.021) and building × design × order × occupation ($F_{1, 148}$ = 3.52, $p = 0.063, \eta_{p}^{2}$ = 0.023). Considering influence of other occupants, a significant building × order × occupation ($F_{1, 148}$ = 5.673, $p = 0.019, \eta_{p}^{2}$ = 0.037) interaction was found, such that office workers were less influenced by other occupants in the second trial, whereas teachers were more influenced by other occupants in the second trial (Fig. 7A). This result indicated that in the second trial, office workers possibly thought more about self-preservation, whereas teachers considered more about the safety of others. There was also a significant design × occupation ($F_{1, 148}$ = 4.775, $p = 0.03, \eta_{p}^{2}$ = 0.031) interaction, such that office workers were more influenced by other occupants in the standard buildings, whereas teachers were more influenced by other occupants in enhanced buildings (Fig. 7B). It is possible that office workers might focus more on others in the standard building because they chose to follow other occupants when evacuating the building. However, when running was less of an option in enhanced buildings, they did not pay as much attention to other occupants’ behavior. Given the complex nature of our experiment design, there was also an inscrutable design × order × occupation ($F_{1, 148}$ = 3.519, $p = 0.063, \eta_{p}^{2}$ = 0.023) interaction (see *Analysis of occupant influence on participants’ response* in the Supplementary Information).

**Figure 8:**
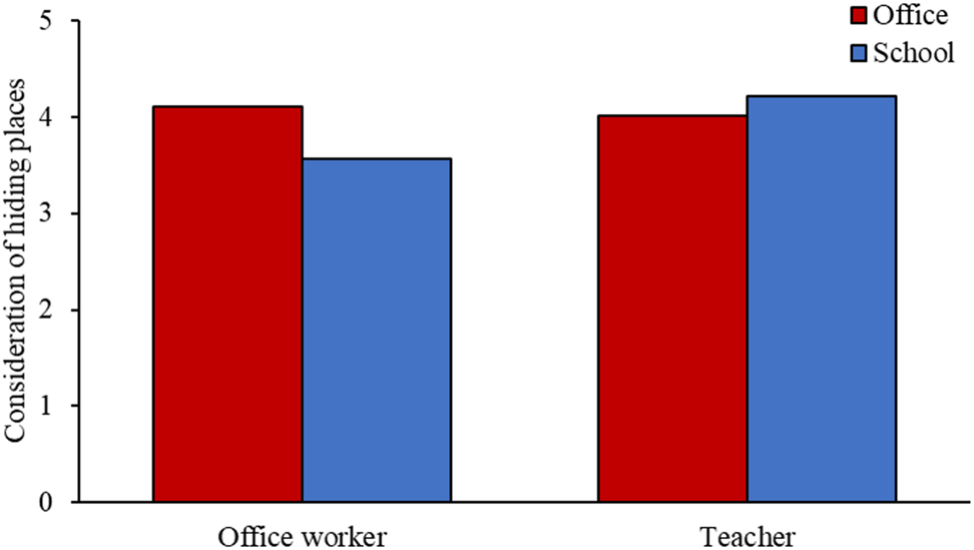
The effect of building × occupation interaction on participants’ consideration of hiding places.

The degree to which participants reported considering other factors (i.e., exits, hiding places, occupant safety) when making their decisions was also analyzed. First, there was a significant building × order ($F_{1, 148}$ = 8.639, $p = 0.004, \eta_{p}^{2}$ = 0.055) interaction on the consideration of exits, such that participants considered exits more in the second trial (M = 4.45) than the first (M = 4.11). This result suggested that participants might have thought more about how not to get trapped in the building in the second trial, after having already experienced the shooting. Given the complex nature of our experiment design, there was also an inscrutable building × design × order × occupation ($F_{1, 148}$ = 4.089, $p = 0.045, \eta_{p}^{2}$ = 0.027) interaction (see *Analysis of participants’ consideration of exits* in the Supplementary Information). Second, there was a significant building × occupation ($F_{1, 148}$ = 10.54, $p = 0.001, \eta_{p}^{2}$ = 0.066) interaction on the consideration of hiding places, such that office workers considered hiding places more in the office, whereas teachers considered hiding places more in the school (Fig. 8). This result suggested that participants were more inclined to stay and hide in a building that matched their occupational context, perhaps because of higher familiarity with the building. Third, even though participants did not have direct interactions (e.g., talking, non-verbal coordination) with other occupants, we found a significant main effect of occupation ($F_{1, 148}$ = 11.192, $p = 0.001, \eta_{p}^{2}$ = 0.07) on participants’ concerns for the safety of other occupants: teachers (M = 3.04) had more concerns for other occupants’ safety compared with office workers (M = 2.42), possibly because their occupation requires supervision and caregiving for others, where office work does not. Again, given the complex nature of our experiment design, there was also an inscrutable design × order × occupation ($F_{1, 148}$ = 5.294, $p = 0.023, \eta_{p}^{2}$ = 0.035) interaction (see *Analysis of participants’ concerns for occupant safety* in the Supplementary Information).

## Discussion

The results of this study revealed that building design played an important role in participants’ responses to active shooter incidents. In enhanced buildings where countermeasures were implemented, participants had a longer response time and fewer decisions of “run.” Indeed, since access control was implemented in enhanced buildings, several exits became inaccessible. Participants who decided to evacuate had to travel a longer distance in enhanced buildings, such as evacuating via Exit 3 at the end of the hallway. In standard buildings, however, the availability of more exits allowed participants to choose a closer exit. This finding was consistent with several previous studies, which demonstrated that people prioritize to choose exits in close proximity during evacuation, as their primary objective is to stay away from emergencies^65,66^. While access control is important for building security, eliminating entry (or exit) points to a building may be detrimental to human safety during active shooter incidents.

Participants in the enhanced buildings also chose to hide significantly more in the kitchen and conference room/teacher’s lounge, possibly due to the decreased ability to evacuate. More hiding decisions in enhanced buildings might also be related to the implementation of frosted windows in the kitchen and conference room/teacher’s lounge, as staying out of shooters’ sight is crucial for survival and is frequently mentioned in emergency trainings^12,64^. In contrast, even though some participants chose to hide under staircases in standard buildings, they were incapable of doing so in enhanced buildings due to the removal of storage rooms to prohibit shooters to hide. This result suggested that hiding is an essential component of people’s responses to active shooter incidents. Yet, hiding behavior has been underexplored, with only a few studies that evaluated hiding behavior using predefined rules (e.g., choose to hide based on the distance to shooters)^13,67^. People should not be assumed to have a sole response to active shooter incidents, and a balance should be achieved between improving building security and providing multiple response options. This result is consistent with a recent study, which demonstrated that a multi-option response plan was more effective than the traditional lockdown approach^7^. Overall, our study revealed that human-building interactions should be taken into consideration to improve human safety, as echoed by several recent studies^1,22,68^.

In this study, building types were different between the two trials. The results revealed that both building types and trial order affected participants’ responses: participants in the school spent a longer time than those in the office. A possible reason for this finding is that participants might be generally more familiar with offices, which facilitated their reading of the building typology and perception of emergency cues^69,70^. Moreover, although unexpected, we found the order of experiencing the office and school had a significant effect on participants’ responses across the two trials. The participants experiencing the office before school had more decisions of “run” in standard buildings, compared with those experiencing the school before office. Similarly, the participants experiencing the school before office had more negative perceptions of the shooter. Such effect of order might be attributed to the consistency of participants’ behavior/perception when experiencing similar scenarios or making multiple decisions^71^. When experiencing the standard office in the first trial, participants’ first intuition might be to evacuate immediately rather than consider the safety of others. Similarly, when experiencing the school in the first trial, participants might perceive the shooter more negatively as students are the victims. Depending on the building context in the first trial, participants’ behavior/perception was carried over to the second trial. Additionally, our results showed that participants had a shorter response time in the second trial. To interpret this finding, we investigated participants’ subjective responses after the second trial and unsurprisingly, we found participants had very high familiarity with the building (M = 4.28 on a scale from 1 to 5) and emergency (M = 4.127). This result indicated that greater familiarity might allow participants to respond more efficiently, which is consistent with previous findings^2^. Such impact of familiarity also suggest that training is an effective approach to improve human safety, as people can be informed about appropriate responses and obtain increased familiarity with different emergency scenarios^72^. Future research could investigate the effectiveness of training for people’s responses during active shooter incidents, especially with an emphasis on innovative training approaches to improve training effectiveness^9^.

With regards to participants’ roles (occupational backgrounds), several interesting findings were identified. First, teachers had significantly more concerns for occupant safety than office workers. Teachers are routinely trained to care for students when an emergency occurs, which might have a direct impact on their responses to active shooter incidents^73^. More interestingly, the responses of office workers and teachers were correlated with the building and social contexts. For instance, even though the virtual occupants had the same appearance in the office and school, office workers had more positive perceptions of occupants in the office, whereas teachers had more positive perceptions of occupants in the school. This finding had to do with participants’ psychological ties with others, as they tended to have closer relationships with familiar people or those sharing common identities^39,74^. Additionally, office workers considered hiding places more in the office, while teachers considered hiding places more in the school. This finding could be attributed to participants’ confidence in the building context: people are usually more confident to shelter in place in a building they are frequently exposed to, whereas if they are in an unfamiliar building, leaving the scene tends to be their first resort^75^. Finally, our results revealed that in contrast to office workers, teachers had more “hide” decisions and were more influenced by other occupants in the second trial. This finding again suggested the impact of participants’ daily roles. For office workers, the main goal is self-preservation. For teachers, on the other hand, they need to consider a duty to care for other occupants in addition to themselves. This is further reflected in participants’ feedback during the experiment: some teachers mentioned that during the first trial, they acted reactively due to the sudden onset of shooting, whereas in the second trial, they considered more the responsibility of being a teacher.

Overall, the answers to our three research questions were all “yes.” First, some building design-related countermeasures aimed for improving building security could affect participants’ response time and choices among run, hide, and fight. Second, participants had a longer response time in the school than office and their response time was significantly reduced in the second trial. Third, office workers had more positive perceptions of other occupants in the office, whereas teachers had more positive perceptions of other occupants in the school. Teachers also had more concerns for other occupants’ safety. Our research findings showed that there is a tradeoff between preventing the occurrence of active shooter incidents and improving occupant safety when an incident occurs. Moreover, the effectiveness of countermeasures can vary in different shooting scenarios, hence appropriate strategies should be adopted given the specific context^76^. For instance, the primary population and function of a building, as well as people’s familiarity with the building should be taken into consideration when developing and implementing countermeasures.

Moreover, our results demonstrated the effectiveness of virtual environments for behavioral studies, especially in situations that would not be safe or feasible to study in person (e.g., building emergencies, proximal threats)^58,77^. That being said, there were still limitations associated with this study. First, while our findings show that people’s roles affect their responses to active shooter incidents, we only recruited office workers and teachers in this study, hence the results may not be generalizable to other population groups. Also, even though we recruited participants nationwide, the results may not apply to all office workers and teachers as other factors, such as their educational levels and previous training experience can affect their behavior as well. Second, while multiple countermeasures were examined in this study, some building attributes (e.g., width of exits and hallways) that depend on macroscopic characteristics of human behavior (e.g., crowd flow rate) cannot be easily examined using virtual experiments. Crowd simulations could be a more appropriate approach for examining these countermeasures, which should be further explored by future research^78^. Third, a countermeasure could be implemented in multiple ways. For example, barriers, vehicles, physical guards, and limiting entrance/exits could all be used for the purpose of access control. In this study, the implementation of countermeasures was based on the results of our literature review and focus group interviews^9,61^. Other forms of implementation could be explored and examined in future research.

## Conclusions

Humans, buildings, and emergencies are three interconnected components. Their relationships should be considered altogether for improving human safety during building emergencies. However, human behavior has not been sufficiently considered when developing and implementing security countermeasures for active shooter incidents. In this study, we conducted virtual experiments to empirically examine the impact of security countermeasures on people’s responses to active shooter incidents, with participants’ occupation as well as building and social contexts taken into consideration. Our findings highlighted that some security countermeasures intended to improve building security may negatively affect people’s response to active shooter incidents. The emergency context and daily roles could also determine how people respond to active shooter incidents. Therefore, a holistic assessment of security countermeasures involving human behavior, roles and emergency contexts is needed to effectively improve human safety.

## Supplementary Information

Supplementary Information.

## Data Availability

The datasets generated during and/or analyzed during the current study are available from the corresponding author on reasonable request.
