# Supplementary material for: The impact of security countermeasures on human behavior during active shooter incidents
Source: Sci Rep. 2022 Jan 18;12:929. doi: 10.1038/s41598-022-04922-8 (PMC8766576; doi:10.1038/s41598-022-04922-8)
Supplement: Supplementary file 1 — Supplementary Information. [file 41598_2022_4922_MOESM1_ESM.docx]

**The Impact of Security Countermeasures on Human Behavior during Active Shooter Incidents**

Runhe Zhu ^1^, Gale M. Lucas ^2^, Burcin Becerik-Gerber ^1,^ *, Erroll G. Southers ^3^, Earl Landicho ^2^

^1^ Sonny Astani Department of Civil and Environmental Engineering, Viterbi School of Engineering, University of Southern California, Los Angeles, CA, 90089, USA

^2^ USC Institute for Creative Technologies, Los Angeles, CA, 90094, USA

^3^ Sol Price School of Public Policy, University of Southern California, Los Angeles, CA, 90089, USA

**Corresponding Author:** Burcin Becerik-Gerber.

**Email:** becerik@usc.edu **Tel:** 213-740-0603

**Address:** 3620 S. Vermont Ave. KAP 210A Los Angeles, CA 90089, USA

**Supplementary information**

*Analysis of participants’ emotional response*

Given the complex nature of our experiment design, there were inscrutable valance × time × design ($F_{2, 296}$ = 9.319, $p<0.001, \eta_{p}^{2}$ = 0.059) and valence × time × occupation ($F_{2, 296}$ = 3.018, $p=0.062, \eta_{p}^{2}$ = 0.02) interactions on participants’ emotional response: the participants experiencing standard buildings (M = 3.13 before the first trial, M = 2.85 after the first trial, M = 2.68 after the second trial for positive emotions. M = 1.47 before the first trial, M = 2.32 after the first trial, M = 2.26 after the second trial for negative emotions) had more significant decrease of positive emotions and increase of negative emotions compared with those experiencing enhanced buildings (M = 2.91 before the first trial, M = 2.86 after the first trial, M = 2.77 after the second trial for positive emotions. M = 1.4 before the first trial, M = 2.03 after the first trial, M = 1.88 after the second trial for negative emotions). Similarly, office workers (M = 3.07 before the first trial, M = 2.84 after the first trial, M = 2.7 after the second trial for positive emotions. M = 1.46 before the first trial, M = 2.28 after the first trial, M = 2.2 after the second trial for negative emotions) had more significant decrease of positive emotions and increase of negative emotions than teachers (M = 2.96 before the first trial, M = 2.86 after the first trial, M = 2.74 after the second trial for positive emotions. M = 1.41 before the first trial, M = 2.07 after the first trial, M = 1.94 after the second trial for negative emotions).

There were also other main effects and lower-order interactions on participants’ emotional response, which were less relevant as they collapsed across time/valence or were qualified by the above effects: there were significant main effects of time ($F_{2, 296}$ = 9.029, $p=0.001, \eta_{p}^{2}$ = 0.057) and valence ($F_{1, 141}$ = 193.325, $p<0.001, \eta_{p}^{2}$ = 0.578), and significant time × design × order ($F_{2, 296}$= 4.365, $p=0.021, \eta_{p}^{2}$ = 0.029), time × design × order × occupation ($F_{2, 296}$ = 3.342, $p=0.049, \eta_{p}^{2}$ = 0.022), and valence × design × order × occupation ($F_{1, 148}$ = 7.861, $p=0.006, \eta_{p}^{2}$ = 0.05) interactions on participants’ emotional response. Moreover, significant order × occupation interaction ($F_{1, 148}$= 4.733, $p=0.031, \eta_{p}^{2}$ = 0.031) was found as a between-subjects effect.

*Analysis of participants’ response time*

Given the complex nature of our experiment design, there was an inscrutable design × order × occupation interaction ($F_{1, 148}$ = 3.149, $p=0.078, \eta_{p}^{2}$ = 0.021) on participants’ response time: the office workers experiencing the office before school spent significantly less time in standard buildings (M = 29.78) than enhanced buildings (M = 47.26). However, the impact of design on response time was not as significant for teachers or office workers experiencing the school before office.

*Analysis of shooter influence on participants’ response*

Given the complex nature of our experiment design, there were inscrutable design × order × occupation ($F_{1, 148}$ = 3.221, $p=0.075, \eta_{p}^{2}$ = 0.021) and building × design × order × occupation ($F_{1, 148}$ = 3.52, $p=0.063, \eta_{p}^{2}$ = 0.023) interactions on influence the shooter had on participants’ response: in the first trial, office workers (M = 4.53 for standard buildings, M = 4.01 for enhanced buildings) were more influenced by the shooter in standard buildings, whereas teachers (M = 4.17 for standard buildings, M = 4.57 for enhanced buildings) were more influenced by the shooter in enhanced buildings. In the second trial, shooter influence was similar for both office workers (M = 4.48 for standard buildings, M = 4.37 for enhanced buildings) and teachers (M = 4.36 for standard buildings, M = 4.42 for enhanced buildings) in standard and enhanced buildings.

*Analysis of occupant influence on participants’ response*

Given the complex nature of our experiment design, there was an inscrutable design × order × occupation interaction ($F_{1, 148}$ = 3.519, $p=0.063, \eta_{p}^{2}$ = 0.023) on influence other occupants had on participants’ response: for those experiencing the school before office, office workers (M = 4 for standard buildings, M = 3.25 for enhanced buildings) were more influenced by occupants in standard buildings, whereas teachers (M = 3.5 for standard buildings, M = 4.03 for enhanced buildings) were more influenced by occupants in enhanced buildings.

*Analysis of participants’ consideration of exits*

Given the complex nature of our experiment design, there was an inscrutable building × design × order × occupation interaction ($F_{1, 148}$ = 4.089, $p=0.045, \eta_{p}^{2}$ = 0.027) on participants’ consideration of exits: office workers (M = 4.03 for the first trial, M = 4.49 for the second trial) tended to be more influenced by exits in the second trial, whereas teachers experiencing standard buildings (M = 4.34 for the first trial, M = 4.29 for the second trial) were influenced by exits approximately the same between the two trials.

*Analysis of participants’ concerns for occupant safety*

Given the complex nature of our experiment design, there was an inscrutable design × order × occupation interaction ($F_{1, 148}$ = 5.294, $p=0.023, \eta_{p}^{2}$ = 0.035) on participants’ concerns for the safety of other occupants: office workers experiencing the office before school (M = 1.95 for standard buildings, M = 2.72 for enhanced buildings) had more concerns for occupant safety in enhanced buildings, which also applied for teachers experiencing the school before office (M = 2.88 for standard buildings, M = 3.4 for enhanced buildings). In contrast, if office workers experienced the school before office (M = 2.66 for standard buildings, M = 2.32 for enhanced buildings) or teachers experienced the office before school (M = 2.98 for standard buildings, M = 2.89 for enhanced buildings), they had more concern for occupant safety in standard buildings.
